# Response and Toxicity to Cytarabine Therapy in Leukemia and Lymphoma: From Dose Puzzle to Pharmacogenomic Biomarkers

**DOI:** 10.3390/cancers13050966

**Published:** 2021-02-25

**Authors:** Raffaele Di Francia, Stefania Crisci, Angela De Monaco, Concetta Cafiero, Agnese Re, Giancarla Iaccarino, Rosaria De Filippi, Ferdinando Frigeri, Gaetano Corazzelli, Alessandra Micera, Antonio Pinto

**Affiliations:** 1 Italian Association of Pharmacogenomics and Molecular Diagnostics, 60126 Ancona, Italy; rdifrancia@iapharmagen.org; 2 Hematology-Oncology and Stem Cell transplantation Unit, National Cancer Institute, Fondazione “G. Pascale” IRCCS, 80131 Naples, Italy; s.crisci@istitutotumori.na.it (S.C.); g.iaccarino@istitutotumori.na.it (G.I.); rdefilip@unina.it (R.D.F.); g.corazzelli@istitutotumori.na.it (G.C.); a.pinto@istitutotumori.na.it (A.P.); 3 Clinical Patology, ASL Napoli 2 Nord, “S.M. delle Grazie Hospital”, 80078 Pozzuoli, Italy; angela.demonaco@aslnapoli2nord.it; 4 Medical Oncology, S.G. Moscati, Statte, 74010 Taranto, Italy; 5 Università Cattolica del Sacro Cuore, 00168 Rome, Italy; agnese.re@unicatt.it; 6 Department of Clinical Medicine and Surgery, Federico II University, 80131 Naples, Italy; 7 UOC Onco-Hematology, AORN SS Anna e Sebastiano, 81100 Caserta, Italy; ferdinando.refrigeri@ospedale.caserta.it; 8 Research and Development Laboratory for Biochemical, Molecular and Cellular Applications in Ophthalmological Sciences, IRCCS—Fondazione Bietti, 00184 Rome, Italy

**Keywords:** Ara-C, pharmacogenetics, target therapy, mechanism of resistance

## Abstract

**Simple Summary:**

In this review, the authors propose a crosswise examination of cytarabine-related issues ranging from the spectrum of clinical activity and severe toxicities, through updated cellular pharmacology and drug formulations, to the genetic variants associated with drug-induced phenotypes. Cytarabine (cytosine arabinoside; Ara-C) in multiagent chemotherapy regimens is often used for leukemia or lymphoma treatments, as well as neoplastic meningitis. Chemotherapy regimens can induce a suboptimal clinical outcome in a fraction of patients. The individual variability in clinical response to Leukemia & Lymphoma treatments among patients appears to be associated with intracellular accumulation of Ara-CTP due to genetic variants related to metabolic enzymes. The review provides exhaustive information on the effects of Ara-C-based therapies, the adverse drug reaction will also be provided including bone pain, ocular toxicity (corneal pain, keratoconjunctivitis, and blurred vision), maculopapular rash, and occasional chest pain. Evidence for predicting the response to cytarabine-based treatments will be highlighted, pointing at their significant impact on the routine management of blood cancers.

**Abstract:**

Cytarabine is a pyrimidine nucleoside analog, commonly used in multiagent chemotherapy regimens for the treatment of leukemia and lymphoma, as well as for neoplastic meningitis. Ara-C-based chemotherapy regimens can induce a suboptimal clinical outcome in a fraction of patients. Several studies suggest that the individual variability in clinical response to Leukemia & Lymphoma treatments among patients, underlying either Ara-C mechanism resistance or toxicity, appears to be associated with the intracellular accumulation and retention of Ara-CTP due to genetic variants related to metabolic enzymes. Herein, we reported (a) the latest Pharmacogenomics biomarkers associated with the response to cytarabine and (b) the new drug formulations with optimized pharmacokinetics. The purpose of this review is to provide readers with detailed and comprehensive information on the effects of Ara-C-based therapies, from biological to clinical practice, maintaining high the interest of both researcher and clinical hematologist. This review could help clinicians in predicting the response to cytarabine-based treatments.

## 1. Introduction

Cytarabine or Cytosine arabinoside (1-β-D-arabinofuranosylcytosine) is a deoxycytidine nucleoside analog renowned among the most effective antineoplastic agents in upfront and salvage programs for myeloid and lymphoid leukemias, as well as Hodgkin and Non-Hodgkin lymphomas [1,2,3,4,5]. Despite forty years of thorough clinical application, treatment advances as a result of on cytarabine-containing regimens have largely lagged. Patients with acute myeloid leukemia (AML) still have their overall survival below 30%, due to both intrinsic and acquired chemotherapy resistance, and lymphoma patients benefit only of temporary disease control, without any definitive cure [6,7,8]. In the meanwhile, the load of severe, life-threatening, or lethal toxicities has remained substantial and mostly unpredictable [9,10,11,12,13,14]. Hence, the optimal efficacy and toxicity trade-off and appropriate clinical settings for cytarabine have remained partway undefined, even with continuous efforts to custom dose scheduling, and stratify prognostic host and tumor characteristics, including cytogenetic and molecular markers. Genetic dissimilarity with large inter-individual differences on pharmacokinetics, as well as pharmacodynamics have been unveiled for various chemotherapeutic agents by pharmacogenetic studies through candidate-genes approaches focusing on drug metabolism. Pharmacogenomics data on genetic and molecular determinants of response were obtained at tumor level by means of newer genome-wide association (GWA) studies. Germline genetic variants have been associated with chemotherapy-induced phenotypes and used to predict toxicity as in the illustrative example of thiopurine methyl-transferase enzyme activity, a major determinant of the activity of the adenine analog 6-mercaptopurine in leukemias and bowel inflammatory diseases. Since the conventional dose of 6-mercaptopurine produces life-threatening toxicity in individuals with certain alleles variants of thiopurine S methyltransferase, the detection of thiopurine S methyltransferase gene mutations is now recommended, and individuals with non-functional alleles can be efficiently treated with reduced doses of 6-mercaptopurine [15].

The metabolism and mechanism of action of cytarabine are directly linked to the biotransformation of its physiological deoxyribonucleotide counterpart, the natural nucleoside deoxycytidine, including membrane transportation, intracellular activation, and interaction with cellular targets. Gene products involved in this process have been well characterized and include transporters of solute carriers (SLC) and ATP-binding cassette (ABC) families, activators such as deoxycytidine kinase (DCK), nucleoside diphosphate kinase (NDK), and ribonucleotide reductase, as well as the catabolyzers of cytidine deaminase (CDA) and 5-nucleotidases [16,17].

Genetic variants for cytarabine-metabolizing enzymes and carriers have been identified, and coding or regulatory polymorphisms have been proposed of functional significance and clinical relevance, so advocating properly devised trials to validate putative associations with phenotypes of toxicity or chemo-resistance [18]. Specific panels of genetic determinants for response and toxicity can be derived for clinical testing, to tailor treatment preventing severe toxicities or diverting treatment in cases of drug-resistance. However, the application of pharmacogenomics to cytarabine treatment is still hindered by the inadequate diffusion and affordability of genotyping methods in routine clinical diagnostics, challenges in statistical validation, and hazy evidence that pharmacogenetics and genomics testing improve patients’ outcomes [19,20]. In the meantime, the understanding of underlying processes of cytarabine intracellular metabolism through bioactivation and detoxication pathways has led to the development and active clinical investigation of new formulations of the drug with optimized pharmacokinetics [21].

In this article, the authors propose a crosswise examination of cytarabine-related issues, ranging from the spectrum of clinical activity and severe toxicities, through updated cellular pharmacology and drug formulations, to the genetic variants associated with drug-induced phenotypes. The goal of this review is to give readers an all-round information on the effects of Ara-C based therapies, from biology to clinical practice. This issue could help oncologist to plan optimal dosage and combination with other drugs to make a personalized treatment.

## 2. Spectrum of Clinical Uses and Serious Toxicities of Cytarabine

The selective action against rapidly dividing cells and the lack of metabolic activation in solid tumors have ensued from broad activity, and focusing the application of hematological malignancies [22]. The current dosage and schedules in Leukemia and Lymphoma are a result of many years of trials, clinical observations, and predictive models [23].

### 2.1. Leukemia

Since 1974, cytarabine is used either alone or in combination with an anthracycline (daunorubicin or idarubicin) in virtually all induction regimens for acute myeloid leukemia (AML), and as a component of consolidation and maintenance programs after remission is attained (Table 1).

The incorporation of cytarabine is needed also in AML subtypes with exquisite susceptibility to anthracycline treatment, such as acute promyelocytic leukemia [24]. As a result of its short half-life and rapid plasmatic inactivation, different schedules and dose-levels of cytarabine have been adopted for intravenous infusion or injection of cytarabine in clinical practice (low, standard, high, and, more recently, intermediate cytarabine doses) while intrathecal administration is regularly employed for prophylaxis and treatment of meningeal leukemia and lymphoma [25,26,27] (Table 1). Although cytarabine is used most commonly in regimens of 100 to 200 mg/m^2^/d for 5 to 7 days, other high-dose and low-dose schedules have been used for treating leukemia. The clinical activity of low-dose cytarabine in AML has been evaluated particularly in older patients or with preexistent myelodysplasia [28,29]. These regimens adopted dosages in the range of 3 to 20 mg/m^2^/d for up to 3 weeks, with the expectation that low doses would produce less toxicity and promote leukemic cell differentiation (or apoptosis). In general, though the low-dose regimens produce less toxicity, in terms of myelosuppression, than previously hydroxyurea-based treatments [30].

The high-dose schemes, usually 2 to 3 g/m^2^ every 12 h for up to six doses, was introduced about three decades ago, and, after the landmark CALGB study published in 1994, it became central to the improvement in the treatment of patients with AML [31,32]. In AML, high-dose cytarabine is used primarily in the consolidation phase [33] and upfront in patients with unfavorable, intrinsically drug resistant, oncogenic subtypes (8;21), inv16, del16, t(16;16) [33]. For the last two decades, high-dose cytarabine has been the optimal post-remission therapy for patients with AML in first remission not proceeding to allogenic transplantation [34,35].

Due to concern of severe side effects, some researchers have recently suggested a possible replacement of high-dose cytarabine with an equally effective less toxic regimen delivering intermediate dosing (1000 mg/m^2^ for each dose), so to increase the therapeutic index of the drug [36,37].

Recently, in patients not eligible for intensive Ara-C dosage, Venetoclax plus a low-dose Ara-C (LDAC) demonstrates clinically meaningful improvement in remission rate and Overall Survivor (OS) vs LDAC alone (#NCT03069352) [38].

Venetoclax is a BCL2 inhibitor, this new class of drugs promising a manageable safety in optimized schedules combined to antimetabolites.

### 2.2. Lymphoma

Beyond the use in myeloid leukemia, cytarabine had a wide and established use, especially through the high-dose regimen, in upfront therapy against very aggressive lymphoproliferative disorders, such as acute lymphocytic leukemia and Burkitt and Burkitt-like lymphomas [52,53,54,55,56] (Table 2). Cytarabine-containing regimens also has exquisite activity in the first-line treatment of mantle cell lymphoma and are going to represent a real new benchmark in this difficult to treat lymphoma subtype [57,58,59].

Notably, high-dose cytarabine has represented, in the last 20 years, a cornerstone of salvage programs for patients with recurrent Hodgkin and non-Hodgkin lymphoma, mostly in combination with platinating agents [60,61,62,63]. In the same setting of patients, it had been also employed for mobilizing peripheral stem cells and incorporated into cytoreductive high-dose therapy before autologous transplantation [64,65] (Table 2). Since a high-dose regimen leads to high drug levels in the cerebrospinal fluid, this regimen may provide adequate prophylaxis for lymphomatous localization and integrate conventional intrathecal administration of cytarabine delivered at a low dose as a single agent or in combination with methotrexate and dexamethasone (Table 2). High dosage cytarabine is also an essential component of systemic therapy for primary lymphomas of the central nervous system (CNS) [66].

### 2.3. Toxicity Profiles

The toxicity profile of cytarabine is highly dependent on the dose and schedule of administration. With a standard 7-day regimen, myelosuppression is dose-limiting. Leukopenia and thrombocytopenia have the raiders occurring between days 7 and 14 after drug administration, with cytopenia duration eventually influenced by the concomitant use of other cytotoxic agents or previous treatment with chemotherapy. Gastrointestinal toxicity usually manifests as a mild-to-moderate mucositis and diarrhea. Occasionally acute pancreatitis has been reported in patients receiving cytarabine as a continuous infusion and in patients treated with L-asparaginase [72,73]. The so-called ‘cytarabine syndrome’ may occur within 12 h after the start of drug infusion with the onset of fever, myalgia, joint and bone pain, maculopapular rash, keratoconjunctivitis, and occasional chest pain [74]. This syndrome most likely represents an allergic reaction to cytarabine because patients usually develop symptoms months after the first dose, and corticosteroids can prevent its onset. Symptoms usually resolve within 24 h when cytarabine is discontinued as in pediatric patients [75].

With the administration of cytarabine at high doses (2 to 3 g/m^2^ with each dose), common major side effects include biphasic pancytopenia, central nervous system toxicity, skin eruptions, and hyperbilirubinemia in more than 10% of patients while infection may affect two thirds of patients contributing to a treatment-related mortality rate of approximately 5% [32,76]. When present, skin eruptions is often followed by fever. Notably, the rate of severe central nervous system adverse events (e.g., somnolence and confusion, and rarely seizures, cerebral dysfunction, acute cerebellar syndrome) is approximately 12% of overall patients, but it rises approximately to 30% in patients over the age of 60, 40% of whom may be left with a permanent disability [77]. Acute cerebellar toxicity manifests as dysarthria with truncal and gait ataxia or less commonly as a cerebral syndrome manifesting as encephalopathy, psychosis, seizures, and coma. The pathogenesis of this syndrome is unknown, but there is widespread loss of Purkinje cells in the cerebellum [78]. The characteristic syndrome begins with somnolence and occasionally an encephalopathy that develops two to five days after beginning treatment. It may also be delayed, occurring up to 3–8 days after treatment has begun. The severe cerebellar toxicity may cause treatment discontinuation in a low subset of patients [78]. High-dose cytarabine may infrequently cause also peripheral neuropathies resembling Guillain–Barré syndrome, brachial plexopathy, lateral rectus palsy, optic neuropathy, or an extrapyramidal syndrome [79,80,81]. Risk factors for neurotoxicity include cumulative cytarabine dose, prior CNS disease and renal impairment (incidence may be up to 55% in patients with renal impairment); high-dose therapy (>18 g/m^2^ per cycle) and age higher than 50 years also increases the risk for cerebellar toxicity [77,82]. Cases of fatal cardiomyopathy had been reported when high-dose cytarabine was used in combination with cyclophosphamide as a preparation regimen for transplantation [83]. Anaphylaxis resulting in acute cardiopulmonary arrest has been reported as well as sudden respiratory arrest syndrome occurring 6 to 12 h following drug administration. With high dose regimen, gastrointestinal toxicity can lead to bowel necrosis and esophagus ulceration, and the rate and severity of pulmonary side-effects are more pronounced, although not clear in their pathogenesis. A “cytarabine lung” is characterized by subacute respiratory failure accompanied by diffuse changes on chest radiographs, and the diagnosis is usually made when other explanations (such as infection) can be excluded [84]. With cytarabine intrathecal administration, severe adverse events are relatively uncommon. A transverse myelitis may occur similarly to those seen with intrathecal methotrexate administration, and rarely, it has been associated with aseptic meningitis, encephalopathy, headaches, and seizures [79,85]. When cytarabine injection in the cerebrospinal fluid (CSF) overlaps with systemic high-dose methotrexate and high-dose Cytarabine intravenously, there is an increased risk of spinal cord toxicity [86].

Ocular toxicity has been observed with high-dose cytosine arabinoside (3.0 g/m^2^ every 12 h). In some cases, patients referred excessive tearing, photophobia, burning ocular pain and blurred vision, and the ophthalmologic examination confirmed the presence of conjunctival injection, central punctate corneal opacities with subepithelial granular deposits, and decreased visual acuity, all treatment-limiting adverse effect of therapy. At molecular level, it has been suggested that the inhibition of corneal epithelial DNA synthesis due to drug dosage and time of drug exposure.

## 3. Clinical Pharmacology and Cellular Metabolism of Cytarabine

Cytarabine is not administered orally because of the high first-pass elimination in the liver and intestinal metabolism due to the presence of CDA that provide a rapid deamination into the inactive metabolite arabinosyl uracil. Once administered intravenously, Ara-C entries into the cells via specific membrane transport proteins [87]. The bulk of cytarabine deamination is thought to occur in the liver, spleen, and kidney, which have very high activities of CDA. Cytidine deaminase is present in plasma, though, at relatively low activity, while is almost absent in central nervous system. Males have a significantly faster clearance than females [88]. The standard or conventional dose, which ranges from 100 to 200 mg/m^2^ daily and is given by intermittent injection or continuous infusion over 5 to 10 days, achieves steady state plasma concentrations generally in the range of 0.1 to 0.5 μM. In the high-dose protocols with dose equal or exceeding 2 g/m^2^, peak concentrations of cytarabine reach 100 μM.

Due to its hydrophilic properties, cytarabine requires transport into the cells and subsequent intracellular metabolic activation through sequential phosphorylation up to the cytotoxic triphosphate active form, which is incorporated into DNA, as false precursor in place of deoxycytidine triphosphate. This results in inhibition of DNA polymerase, chain termination and stalling DNA and RNA synthesis with the consequent blockage of the cell cycle from G_1_ to the S phase and neoplastic cell death (www.kegg.jp/kegg-bin/showpathway?hsa00240+4830 (accessed on 13 January 2021)) [89,90].

The different phases of drug uptake, activation, and deactivation, are described in detail after wards and synoptically resumed in Table 3 together with key features of cytarabine pharmacology.

### 3.1. Drug Uptake

**Figure 1:**
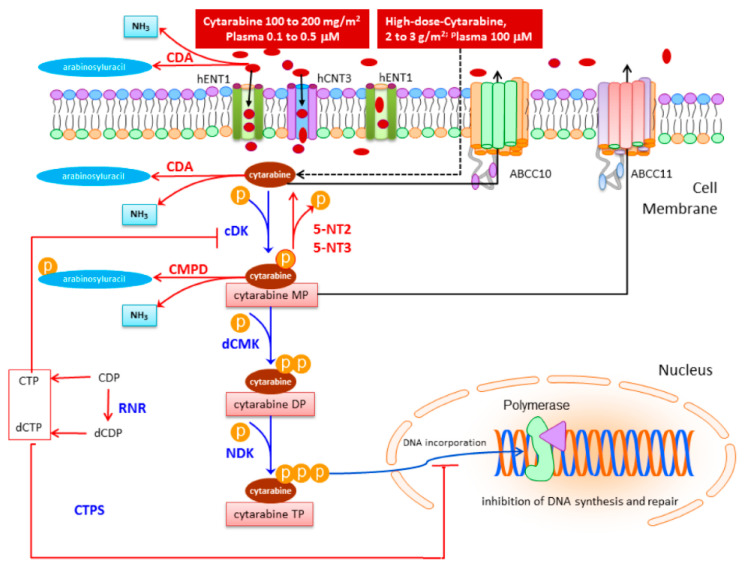
As with other nucleoside analogues and their physiologic counterparts, cytarabine enters cells via nucleoside transport proteins, the most important one being the equilibrative inhibitor-sensitive (es) receptor (ABC). Once inside the cell, cytarabine requires activation for its cytotoxic effects. The first metabolic step is the conversion of cytarabine to cytarabine monophosphate by the enzyme deoxycytidine kinase (DCK). Cytarabine is subsequently phosphorylated to cytarabine diphosphate and cytarabine triphosphate, respectively. Cytarabine triphosphate is a potent inhibitor of DNA polymerases, which, in turn, interferes with DNA chain elongation, DNA synthesis, and DNA repair. In addition, cytarabine is incorporated directly into DNA and functions as a DNA chain terminator, interfering with chain elongation. Catabolism of cytarabine involves two key enzymes, cytidine deaminase (CDA) and deoxycytidyne monophosphate deaminase (DCMD). These breakdown enzymes convert cytarabine and cytarabine monophosphate into the inactive metabolites, uracil arabinoside and arabinosyluracil monophosphate, respectively. Other catabolic enzymes that may affect cytarabine metabolism include pyrophosphatase, 5-nucleotidase. The balance between intracellular activation and degradation is critical in determining the amount of drug that is ultimately converted to cytarabine triphosphate and, thus, its subsequent cytotoxic and antitumor activity.

Cytarabine gains entry into cells primarily as a false substrate through specialized nucleoside transporter proteins of SLC family, the human equilibrative nucleoside transportershENT1 and hENT2 (encoded by the gene *SLC29A1* and *SCL29A2,* respectively) [91,92,93,94] and the human concentrative nucleoside transporters hCNT3 (encoded by the gene *SLC28A3*) [95,96,97,98]. Uptake and accumulation of cytarabine is also regulated by transmembrane transporter proteins of the ABC family, also called human multidrug resistance-associated protein (MRP) family, namely ABCC10 (MRP7) and ABCC11 (MRP8) specifically committed to efflux of deoxynucleotides inactive metabolites and to temper intracellular pools of phosphorylated deoxynucleotides (Figure 1) [99,100].

The integral drug uptake depends on the proper balance of the nucleoside transporters and drug efflux proteins presented on cellular membranes. Therefore, the drug accumulation may be substantially reduced when the expression of hENT1 transporter is deficient, or the activity of ABC drug efflux transporter proteins is elevated.

Cytarabine influx into the cells is strongly correlated with the cell surface abundance of hENT1 transporters [101], so that these membrane proteins are pharmacological determinants for drug bioavailability and response to treatment [102]. The expression of hENT1 can be regulated by the presence on the hENT1 promoter of hypoxia inducible factor 1 (Hif-1) binding sites [103] and according to more recent research, by the nuclear transcription factor peroxisome proliferator activated receptorα (PPARα) [104]. Reduced hENT1 expression and activity has been related with unfavorable therapeutic outcomes in patients with acute myeloid leukemia treated with cytarabine [105,106].

At drug concentrations above 10 µmol/L, the pump-mediated transport process becomes saturated, so that further entry of cytarabine can takes place freely by passive diffusion [107,108].

### 3.2. Drug Activation

Once inside the cells, as shown in Figure 1, activation of cytarabine occurs by means of the step wise *de novo* synthesis of 5′-mono-, di-, and triphosphate derivatives throughout the sequential action of deoxycytidine kinase (DCK), deoxycytidine monophosphate kinase (dCMK), and nucleoside diphosphate kinase (NDK) encoded by the *NME1* gene [94,109]. Deoxycitidine kinase plays a pivotal role since phosphorylation of cytarabine preserves intracellular retention of the drug and prevents from inactivation to its uridine derivative, uracil arabinoside, by cytidine deaminase. The intracellular accumulation of cytarabine triphosphate, the active cytotoxic metabolite, is proportional to the cellular DCK level which has led to the conclusion that DCK enzyme retains a rate-limiting role for the activation of cytarabine [110]. Phosphorylated cytarabine metabolites interfere with the cellular pool of natural nucleosides, are incorporated into DNA and inhibit DNA synthesis in a competitive fashion [111]. Phosphorylated cytarabine metabolites interfere with the cellular pool of natural nucleosides, are incorporated into DNA and inhibit DNA synthesis in a competitive fashion [111]. This inhibition may be synergized with co-administration of others antimetabolites, such as fludarabine and cladribine. In particular, the Km of cytarabine triphosphate and physiological deoxycytidine triphosphate (dCTP) for DNA polymerase are in the same range, so that cytarabine-triphosphate can act as a weak competitive inhibitor of DNA polymerase [112,113]. After incorporation into DNA, cytarabine triphosphate behaves as a relative chain terminator and both replication and DNA repair are inhibited [114].

In vitro studies have revealed that the intracellular concentrations of cytarabine-triphosphate are higher in cytarabine sensitive cells than in resistant cells [109,115]. The retention of cytarabine triphosphate appears to be a critical factor in the response of patients to cytarabine treatment and a correlation between cytarabine triphosphate retention and the duration of complete remission were observed in AML [116]. One of the reasons for giving high-dose cytarabine treatment is to improve the cytarabine triphosphate formation and intracellular cytarabine-triphosphate levels [117]. Increased levels of cytarabine-triphosphate correlate positively with clinical response after high-dose cytarabine treatment [17]; however, Plunkett et al. showed that cytarabine triphosphate formation in leukemic blasts during cytarabine treatment is saturated at plasma levels reached at a dose of 0.5 to 1 g/m^2^, which is considerably lower than a standard high dose of 3 g/m^2^ cytarabine (see the section on clinical pharmacology) [118].

In addition to its activation to cytarabine triphosphate, cytarabine is converted intracellularly into minor metabolites such as cytarabine diphosphocholine, an analog of the physiologic lipid precursor cytidine diphosphocholine that may interfere in lecithin and sphingomyelins synthesis, and be responsible for neurological toxicities [119,120].

Biological activity of cytarabine depends not only on the intracellular concentrations of cytarabine triphosphate but also on the endogenous nucleotide pools of cytidine-triphosphate (CTP) and dCTP. Cytarabine-triphosphate competes with dCTP for incorporation into DNA and to a lesser extent, with CTP for incorporation into RNA. In particular, high levels of dCTP result in resistance to cytarabine due to competition with cytarabine triphosphate for incorporation into DNA. This mechanism may be attractive for combinational administration of others antimetabolites such as fludarabine and cladribine [121]. Hence, the concentration ratios of cytarabine triphosphate versus dCTP and cytarabine triphosphate vs CTP could be expected as predictive therapeutic efficacy [122]. Beyond the contest for incorporation into DNA, dCTP may affect cytarabine metabolism through feedback inhibition of DCK [123,124] and allosteric activation of CDA enzyme with the subsequent weak phosphorylation and enhanced deamination, respectively [125].

Finally, the intracellular levels of dCTP are regulated by the activities of the cytidine triphosphate synthetase (CTPS), a key enzyme in pyrimidine biosynthesis catalyzing the conversion of uridine triphosphate to CTP, and also modified by ribonucleotide reductase (RR), an enzyme composed of a dimerized large (RRM1) and small (RRM2) subunits catalyzing the reduction in ribonucleotides to deoxyribonucleotides for DNA synthesis [126,127]. Activation or inhibition of RR can be directly associated with resistance or sensitivity to cytarabine as tested in a clinical trial [128]. Nucleoside analogs such as fludarabine and cladribine act as inhibitors of RR after intracellular conversion to their deoxynucleotide di- or triphosphate metabolites. Since the inhibition of RR may enhance the cytarabine triphosphate accumulation, the use of agents such as fludarabine and cladribine has been incorporated into therapeutic regimens for AML to potentiate cytarabine metabolism through a sort of biochemical modulation. The same rationale has been applied to enhance cytotoxic activity of cytarabine in lymphocytes of patients with chronic lymphocytic leukemia [129,130,131].

### 3.3. Drug Deactivation

Due to the similarity with the natural precursors required for cellular homeostasis, cytarabine and its metabolites are suitable substrates for various cellular enzymes, which catalyze their conversion into related inactive derivatives. The route of cytarabine inactivation is included in Figure 1.

So, DCK activating function is reversed by cytosolic enzymes belonging to the family of 5′-Nucleotidase (NT5), namely NT5C2 and NT5C3. Opposing dCK, these enzymes catalyze the dephosphorylation of the monophosphate intermediate back to cytarabine through removal of 5′ phosphate [5,132,133].

Also opposing the activation pathway are the two deaminase CDA and deoxycytidine monophosphate deaminase (dCMPD). Cytidine deaminase is a multi-subunit enzyme involved in the maintenance of the pyrimidine nucleotide pool within the cell and physiologically catalyzes the hydrolytic deamination of cytidine to uridine and deoxycytidine to deoxyuridine [109,134]. In cytarabine biotransformation, CDA removes the amine group from its cytosine and converts the drug into the inactive uracil arabinoside derivative. Similarly, CMPD deaminates cytarabine-monophosphate to arabinosyl-uracil-monophosphate [109,134]. A crucial role for this latter enzyme has been suggested in the metabolism of cytarabine-monophosphate in T-lymphoblastic leukemia [17,87,135,136,137].

The balance between enzymatic activation and deactivation determines the effective amount of drug converted into the active metabolite cytarabine-triphosphate. This enzymatic balance varies greatly among cell types and maturity. The kinase deaminase ratio averages 0.03 in human AML, whereas the enzyme activities are approximately equal in acute lymphoblastic leukemia and Burkitt lymphoma [109]. Increased levels of CDA are accounted to play a key role in the development of resistance to cytarabine; in contrast, low activity in CDA enzyme can be related to various toxicities [138].

### 3.4. Mechanisms of Resistance within the Cytarabine Pathway

Studying the pathways involved in the transport, activation, or degradation of cytarabine has allowed the identification of mechanisms of resistance. The foregoing considerations over cytarabine metabolism and transport makes it clear that a number of factors could negatively affect response to cytarabine through the reduction in intracellular levels of the cytotoxic triphosphate metabolite of cytarabine. The mechanisms that critically determines resistance to cytarabine appears to be related to a deficiency of cytarabine cellular uptake and retention, overexpression of enzymes inactivating cytarabine, increased cellular dCTP pools, and increased DNA repair. Synoptically, the mechanism of resistance within cytarabine pathway are resumed in Table 4.

## 4. Genetic Determinants of Response and Toxicity to Cytarabine

### 4.1. Old and Novel Approaches to the Discovery of Pharmacogenomics Markers

The approach to identify genetic variants associated with a cytarabine response phenotype or a serious adverse event was preferentially via a candidate gene or pathway centric approach. Studies on candidate genes have focused on sequence variation, alternative splicing and, above all, expression in neoplastic cells of key cytarabine pathway genes, in particular *SCL29A1*, *DCK*, *CDA*, and *NT5C2* (Table 5). These studies have unveiled that mRNA expression in these genes is influenced by Single Nucleotide Polymorphisms SNPs in their regulatory regions, while SNPs in coding regions could produce amino acid changes capable to affect the protein or activity of the respective genes [17,139,140].

After the advent of the Human Genome Project together with the avowed completion of the human genome sequence [141] and the genomic characterization [142], the application of molecular technologies to interrogate the entire genome has led to the affirmation of GWA studies and to the discovery of more variants. In less than five years the GWA studies have led to new discoveries about genes and pathways involved in cytarabine metabolism and provided a wealth of new biological insights, and findings of potential clinical utility for prognosis or treatment. An odds ratio of 3.0, or even 2.0 depending on population allele frequencies, would be robust to stratify a population. Odds ratio detected by GWA that are below 1.5 can be frequently be explained by cryptic population stratification, regardless of the *p* value associated. Such studies have taken into account pharmacodynamic and pharmacokinetic genes, and considered drug response as determined by multiple genes, also out of the drug metabolic pathway, often contributing smaller effects. Differently from candidate gene approach, which started from the patent knowledge of cytarabine biotransformation pathway, and so was hypothesis driven, GWA approach generated new hypotheses and a mess of new data [143]. However, factors such as inadequate sample size, weak genetic effects and overflowing comparisons made using data on SNPs or expression across the genome predispose GWA approach to false positives and upwardly biased effect sizes with large standard errors especially among SNPs with low minor allele frequencies [144].

These shortcomings and the necessity to replicate and validate GWA data into large cohorts of individuals affected by the same neoplasm and receiving the same cytarabine-including regimen and dose schedule, have prompted researchers in the last years to return to cell-based models [143]. In this respect, the use of human Epstein–Barr virus-transformed lymphoblastoid cell lines (LCLs) has progressively developed as a favorable model system [5,154], also owing to the publicly accessibility of panels with genome-wide genotype and gene expression data, including next-generation sequencing (DNA and RNA-Seq) data, for hundreds of established LCLs [155]. The three main collections of LCLs that have been used in pharmacogenomics research on cytarabine are large Centre d’Étude du Polymorphisme Humain (CEPH) pedigrees, Human Variation Panel populations, and International HapMap Project populations [156,157]. Notably, the International HapMap Project has developed a human haplotype map cataloging the common patterns of DNA sequence variation across world populations through additions of SNP genotypes, phased haplotypes, and linkage disequilibrium information, as well as many samples with the complete genome sequenced to capture additionally common and rare variants. All these data have been regularly updated and enlarged over time, and can be accessed and downloaded from the HapMap Project and SNP Consortium Linkage Map Project websites [158,159,160].

Despite a single-model system cannot signify the complexity of drug effects in the human body, LCLs have the advantages of the easy experimental manipulation and the absence of the in vivo confounders existing in clinical samples. To further enhance evidences from LCLs model and curtail potential in vitro confounders, SNP and gene associations identified within the LCLs model were replicated in relevant tissue and clinical populations. This combined usage of data from both LCLs model and clinical trials has conferred compelling evidence to genotype–phenotype associations and strengthen the findings [155]. Cell models other than LCLs, and including fibroblast cells and peripheral blood mononuclear cells (PBMCs) have also been employed in pharmacogenetic research on cytarabine though with smaller catalogs of lines and accessibility to genetic information.

Cell lines have also been used for functional validation by means of silencing RNA-mediated gene silencing and recombinant protein expression strategies, discoveries from GWA studies that may have resulted in some false positives [5,150,152].

LCLs have been applied successfully also to the study of genetic regulation of gene expression within the causal pathway between genetic variants and the complex phenotypes of toxicity and efficacy. The genomic regions regulating quantitative expression differences, or expression quantitative trait loci (eQTL), have been mapped using 14 CEPH pedigrees and found that gene expression is heritable [161]. Expression levels across the genome can be examined through microarray technology simultaneously, and eQTL studies can integrate genetics and pharmacologic phenotypes, as in the so called ‘triangle method’. After its first presentation in 2007 [162], this type of GWA approach has been effectively adopted to identify novel genetic variants able to predict sensitivity to cytarabine, as well as a variety of chemotherapeutics, including etoposide, cisplatin, carboplatin, and daunorubicin. It develops through the three different arms of (1) preliminary evaluation of relevant associations significant between SNPs and sensitivity to the drug, (2) eQTL analysis performed from this list of SNPs to discover the subset of SNPs linked to the expression of transcripts, (3) assessment of the expression of the list of target genes for significant linear correlation to drug sensitivity. It has been used to compare the pharmacogenomics of cytarabine susceptibility between the HapMap Phase I/II CEU and YRI populations and to explore in LCLs from 60 CA, 54 AA, and 60 HCA individuals of the Human Variation Panel the pharmacogenomics of cytarabine and that of the congener chemotherapeutic agent gemcitabine [98,163]. Population-specific pharmacogenetic signatures consisting of four SNPs clarifying 51% of the variability in cytarabine cytotoxicity were identified among CEU, as well as five SNPs explaining 58% of the variation among the YRI [163].

The results from these studies on cytarabine and from other LCL-based pharmacogenomics research, are contained within the Pharmacogenomics, And Cell database (PACdb, www.pacdb.org (accessed on 13 January 2021)) which provides a unique resource to the researchers [164]. Investigators can look for specific genes and SNPs of interest to determine if they have been found to associate with a particular drug phenotype. At present, PACdb contains summary results for SNP genotype versus cytotoxicity and gene expression versus cytotoxicity for daunorubicin, etoposide, cisplatin, carboplatin, and cytarabine. PACdb also enfolds population-differential expression data and splicing-index data.

Beyond GWA studies, different data generation techniques can be used, depending on the scientific questions and hypothesis under testing. Therefore, if it is assumed that rare, coding variants will be most relevant for the pharmacogenomics trait of interest, exosome sequencing or exome chips would be the most likely methodology chosen. On the contrary, if gene expression variation from eQTL or epigenetic variation are conjectured to be most important, next generation sites (methyl-seq) may be selected instead.

Hereafter, we run through studies addressing genetic variants, polymorphisms, enzyme activities and protein expression levels associated to cytarabine. These are also recapped in Table 4, together with their associated phenotypes.

### 4.2. Drug Uptake/Efflux

#### 4.2.1. Genetic Variants of Cytarabine Transporters

Cytarabine is typically hydrophilic and, unless given at high dose, requires nucleoside transporter proteins to uptake efficiently inside the cells. At conventional doses of 100–200 mg/m^2^ of cytarabine, hENT1 is accounted for up to 80% of cytarabine inward transport though considerable interpatient variations have been observed [165]. hENT1 (*SLC29A1*) coding region appears to be highly conserved and, at functional characterization, genetic variants do not contribute to inter-individual differences in response to nucleoside analog drugs [166,167]. In contrast, in the hENT1 5′UTRpromoter region three out of four naturally occurring haplotypes of three polymorphisms (−1345C>G, −1050G>A, and 706G>C) were associated with higher mRNA expression [145]. Since no functional coding SNPs seem to modulate the function of hENT1 while the variability in expression of hENT1 is reported to affect sensitivity to cytarabine, a possible role of interacting transcription factors may be contributive. So, any genetic variation in the hENT1 locus that may disrupt or create binding sites for Hif-1 and other transcription factors could eventually alter hENT1 expression as well as activation of PPARα [103,104]. Indeed, 30 novel polymorphisms in both coding and in the region of promoter were identified in *SLC29A1* from Japanese subjects; yet the functional significance of these variants remains to be defined [168].

The hENT1 transporter was highly up regulated in biphenotypic leukemia associated with the 11q23 MLL gene (4;11) translocation [169].

Inter-temporal marks variability of hENT1 staining intensities was documented in the Reed–Sternberg cells of Hodgkin lymphoma using immunohistochemistry applied to frozen tissues [170]. Immunochemistry assays for hENT1 was correlated with clinical outcome in CALGB 59804 multicenter trial in Hodgkin lymphoma treated with the cytarabine congener gemcitabine; in this study, the opposite of the logical expected result was found since patients with high hENT expression had a lower likelihood of response and failure-free survival [171,172].

In a study conducted in 115 non-Hodgkin lymphoma patients, a relatively high frequency of hENT1 protein positivity (i.e., immunostaining in >50% of neoplastic cells) was found in malignant follicular center cells and in particular in Burkitt Lymphoma (63%), diffuse large B-cell lymphoma (DLCL; 45%), and follicular lymphoma (40%) [173].

The application of GWA studies to samples from European AML adult patients identified the SNP (rs11140500) in *SLC28A3* for hCNT3 as of clinical relevance [96].

Consistent with the process of an efflux pump and contributive to cytarabine drug resistance, the expression of the ABC transporter *ABCC10* (MRP7) in HEK293 cells reduced the accumulation of cytarabine [100] while *ABCC11* (MRP8) determined increased cellular efflux of phosphorylated cytarabine metabolites [174].

#### 4.2.2. Transporters and Response and Toxicity to Cytarabine

There is a substantial clinical evidence indicating that the efficiency of intracellular cytarabine concentration mediated by hENT1 membrane facilitating diffuser is related to clinical outcome [102].

The cell surface abundance of nucleoside transporter sites is closely correlated to cytotoxicity of cytarabine [101], and a threshold level of hENT1 protein expression is necessary to make cancer cells sensitive to cytarabine [95].

The reduction in hENT1 expression was a common mechanism for resistance to antimetabolite treatment [175,176] and represents a major factor in cytarabine resistance in leukemic blasts of childhood AML [106]. Among 77 AML patients, those presenting hENT1 deficiency at diagnosis had significant worse disease-free survival and overall survival [107]. A high sensitivity to cytarabine, found in children affected by gene rearranged biphenotypic acute lymphoblastic leukemia, has been attributed to highly upregulation of the hENT1 transporter [169].

A study using a cytarabine resistant CCRF-CEM cell line reported that genetic mutations of hENT1 that alter mRNA splicing and protein translation provide mechanisms for resistance to drug treatment [177]. Additional investigation on the genetic basis for the cytarabine resistance of CCRF-CEM leukemia cells led to the discovery of missense mutations in critical amino acids for hENT1 nucleoside recognition and uptake [178].

There is no clear functional impact of the genetic variant in the promoter region of *hENT1* gene, (−706G>C). Some authors reported, anonymously, a statistically significant tendency towards higher mRNA levels in PBMCs from individuals heterozygous for this variant, as compared with wild-type carriers [145]. Others did not find any impact on toxicity and suggest a small part of hENT1 genetic variations in the modulation of cytarabine toxicity in normal blood cells [16]. These latter results are in line with the findings from GWA studies on LCLs [98].

Of significant practical implication is the fact that hENT1 is strongly inhibited by various receptor tyrosine kinase inhibitors, an interaction that could limit cytarabine use with targeted drugs [179,180].

Several studies have shown that the nucleoside transporter hCNT3 is involved in cytarabine cytotoxicity and resistance [95,97]. The SNP (rs11140500) in *SLC28A3* for hCNT3 was recently found to be associated with disease-free survival at multivariate analysis among 154 European AML adult patients on high-dose cytarabine [96]. However, since this SNP represents an uncommon variant, any correlation with clinical and survival data should more properly rely on additional functional studies or replication in larger sample size.

No significant gene polymorphisms have been associated with resistance to cytarabine for ABC transporters, though for *ABCC11* gene SNP (538G>A) has been suggested to be a clinical biomarker for prediction of chemotherapeutic efficacy in breast cancer [181] and SNP (G>A, T546M) predictive for 5-fluorouracil-induced severe toxicity [182].

Recently, a differential expression of *ABCC11* (MRP8) mRNA has been demonstrated in AML blasts from 50 patients, together with a low probability of overall survival at 4 years for those with high expression of MRP8. The result was statistically significant at regression analysis and independent of the dosage of cytarabine delivered during antileukemic treatment [99].

## 5. Drug Activation

### 5.1. Genetic Variants of Kinases

Among the kinases participating to the multistep activity that lead to phosphorylation of cytarabine up to its triphosphate active metabolite, DCK is the rate-limiting enzyme. It exerts highest activity during the S phase of the cell cycle and is strongly inhibited by the physiological dCTP substrate [183].

The gene coding for DCK and a mutated version found in cytarabine resistant cells have been cloned [177,184]. The *DCK* gene is fairly conserved in Caucasians, in contrast to African populations [146] where higher level of *DCK* mRNA expression has been demonstrated [147]. This may explain, to some extent, population differences observed in the grade of sensitivity to cytarabine since higher *DCK* expression could lead to increase intracellular cytarabine triphosphate resulting in increased cellular sensitivity. The activity and expression of *DCK* varies widely in normal and malignant cells; furthermore, there is a 50-fold variation in *DCK* mRNA expression in patient leukemic cells, a 35-fold variation in DCK mRNA in primary AML cells, a 36-fold change in liver tissue and a 150-fold change in human liver metastases of colorectal cancer origin [185].

Several genetic variants in the *DCK* gene have been evidenced: five regulatory mRNA expression SNPs (−125G>T, −201C>T, −289T>A, −360C>G, and −740G>C) and three non-synonymous coding changes Ile24Val, Ala119Gly, and Pro122Ser [147].

The second phosphorylating enzyme, CMPK, is found in more than a hundred-fold higher concentration than DCK. Its affinity for cytarabine-monophosphate is low but greater than the affinity for the competitive physiologic substrate deoxycytidine-monophosphate. Because of its relatively poor affinity for cytarabine-monophosphate, this enzyme could become rate limiting at low cytarabine-monophosphate concentrations [109]. No major polymorphism in CMPK enzyme with clinical application has been identified.

The third phosphorylating enzyme, NDK, appears not to be rate limiting because it is present in very high concentration [109]. In total, five SNPs within the NME1 gene coding for NDK have been found in a retrospective study on 360 Caucasian patients suffering from AML [148].

### 5.2. Kinases and Response and Toxicity to Cytarabine

Clinical studies have shown that low intracellular concentrations of the chemotherapeutic cytarabine in leukemia cells predict poorer outcome to treatment [186,187]. Likewise, low mRNA levels of *DCK* in blast cells predict shorter disease free survival, as well as overall survival in an AML population treated with cytarabine [188]. Ex vivo models using LCLs were able to associate these two observations to SNPs in *DCK*. Examination of LCLs from HapMap populations determined that SNPs within *DCK* resulted in altered enzyme kinetics, increased cytarabine triphosphate intracellular concentrations, higher basal levels of *DCK*, and increased sensitivity to cytarabine [147,163]. Additional SNPs in the 3_untranslated region of *DCK* (positions _36113 and _35708) were also associated with DCK basal expression and cytarabine sensitivity in the HapMap cell lines and cytarabine triphosphate levels in leukemic cell samples from patients with AML, respectively [147,163]. Thus, for cytarabine pharmacogenomics, clinical studies were successful in identifying a biomarker (cytarabine triphosphate levels), and candidate gene (*DCK*), whereas cell-based models identified candidate SNPs associated with these phenotypes that could be potentially useful in clinical dosing algorithms.

The two regulatory SNPs (−360C and −201C) have been in linkage disequilibrium, and have been associated with a more favorable 2 years disease free survival in 122 AML Asian patients, and this was explained by lower DCK mRNA expression leading to reduced transcriptional activation when compared with the −360C/−201C haplotype [189]. These polymorphisms have low frequency in Caucasian population [190]. A reduced enzyme activity in *DCK* 122Ser (rs6743726) reported as being 43 ± 4% of the wild type (WT) allele in vitro in *DCK* coding region [168]. Some authors have found this association to be statistically significant, while others have failed to find it [147,191]; anyway, more recently this polymorphism has been associated with a reduction in enzyme activity as compared with WT that reached statistical significance [192].

In total, five SNPs within the *NDK* gene promoter have been correlated with drug-induced toxicity, in Caucasian AML patients. No significant correlation between SNPs and disease-free survival or OS has been found, but significant correlation with low platelet count has been identified for the *NDK* promoter SNP-835 C/T (rs2302254). In addition, increased risk of neurotoxicity for the SNP-835 C/T for allele T/T has been identified also at multivariate analyses [148].

### 5.3. Genetic Variants of Ribonucleotide Reductase and Cytidine 5′-Triphosphate Synthetase

Another family of ‘activators’ could be represented by RRM1 and RRM2 catalyzing the reduction in ribonucleotides to their corresponding deoxy-ribonucleotides. Diphosporilated cytarabine influence indirectly pharmacokinetics by inhibition of ribonucleotide reductase (RRM1, RRM2, and RRM2B) enzymes that catalyze reactions which generate deoxynucleoside triphosphates required for DNA synthesis.

Low levels of deoxycytidine triphosphate due to CTPS inhibition or inhibition of ribonucleotide reductase lead to Ara-C phosphorylation (by reducing feedback inhibition of DCK), as well as incorporation of Ara-CTP into DNA. Conversely, cytarabine-triphosphate exerts only a weak inhibition and this lack of feedback allows accumulation of the cytarabine nucleoside to a higher concentration [17].

Inhibition of CTPS with cyclopentenyl cytosine has been shown to deplete CTP and dCTP pools and increased sensitivity to cytarabine in human T-cell lymphoblastic cell lines and myeloid leukemia cell lines [126,193,194]. Furthermore, the dNTPase SAMHD1, which regulates dNTP homoeostasis antagonistically to ribonucleotide reductase (RNR), limits Ara-C efficacy by hydrolyzing the active triphosphate metabolite Ara-CTP. Thus, targeting SAMHD1 to enhance Ara-C efficacy is a rational strategy to improve survival in AML and other hematological malignancies [195].

### 5.4. Ribonucleotide Reductase, Cytidine 5′-Triphosphate Synthetase and Response and Toxicity to Cytarabine

Ribonucleotide reductase regulates dCTP and other deoxyribonucleotides levels inside the cells so that its activity is straightly associated to sensitivity or resistance to cytarabine. Inhibition of RR can culminate in the accumulation of cytarabine triphosphate [127]. Biochemical modulation of cytarabine by nucleoside analogs such as fludarabine and cladribine has been shown to be feasible in adult and pediatric leukemia patients as they stimulate the cytarabine triphosphate accumulation by the inhibition of RR [129,196,197].

SNPs within the RRM1 and RRM2 genes were evaluated in the HapMap LCLs from CEU and YRI panels to find possible correlation with expression and cytarabine chemosensitivity. SNPs of expected importance were further evaluated in leukemic blasts from 276 AML patients. RRM1 SNP rs1042919 (which occurs in linkage disequilibrium with various other SNPs) and promoter SNP rs1561876 were related to intracellular cytarabine triphosphate levels, response after up front treatment, disease and overall survival [149].

Consistent with previous studies showing that SNPs in RRM1 are associated with response or toxicity to gemcitabine-based chemotherapy in lung and breast cancer patients [198,199]; findings from a recent study conducted with GWA studies in 154 European and 125 non-European AML adult patients suggest that the SNPs in RRM1 are associated with AML response to chemotherapy that include cytarabine [96]. These results suggest that SNPs within RR retain clinical significance and might represent useful predictive markers of response to cytarabine deserving further validation in larger cohorts.

As regards CTPS, mutations have been identified within the coding region in cytarabine resistant strains of Chinese hamster ovary cells, but none of these mutations was identified in samples from 36 patients, with acute leukemia refractoriness or recurrence [200,201]. Thus, mutations in these sites within the human CTPS gene do not performance a primary mechanism of resistance to cytarabine. In the International HapMap Project database, several SNPs are indicated, and two synonymous SNPs, Gln167Gln and Val500Val were observed with the allele frequency of 0.28 and 0.25, respectively. Future efforts will address the clinical relevance of these findings.

## 6. Drug Deactivation

### 6.1. Genetic Variants of Deaminases and 5′-Nucleotidases

Cytarabine catabolism is performed through processes of deamination, involving CDA and CMPD enzymes, and dephosphorylation by NT5C family enzymes.

The primary role in cytarabine degradation and detoxification is played by CDA.

In total, three important variants in the 5′ UTR promoter region of *CDA* gene (−451C>T, −92A>G, and del-31) were associated to alterations in putative transcription factor binding sites and impacted CDA enzyme activity as assayed in peripheral blood mononuclear cells (PBMCs) of healthy individuals [202]. Of particular relevance is the *CDA* nucleotide variant 79A>C which leads to a non-synonymous amino acid substitution in exon 1 from a Lysine to a Glutamine (Lys27Gln) and results in decreased activity of CDA with a 30% reduction in the cytarabine deamination rate [203]. The nucleotide 79A>C is prevalent in all of the population groups studied, with a 20% frequency of the variant allele in the Japanese and 36% in the European population [204]. The expression of this *CDA* Gln variant has been subsequently assayed through an haplotype analysis in an ethnically diverse sample set [188]. In this study, the silent variant 435C>T (Tyr145Tyr) was reported as frequent and so proposed for integrating the coding variant Lys27Gln and the three promoter variants, −451C>T −92A>G, and −31del, to define, finally, 15 different haplotypes having a frequency >1% among Caucasian population. This set of haplotypes encompasses a wide array of genotypically characterized profiles of toxicity ranging from CDA*1A to CDA*2A, this latter constituted by a combination of variant alleles −451T, −92G, −31Del, and 79C (27Gln), having the least and the most severe toxicities, respectively (Supplemental Table S1) [205]. Recently, a CDA protein-stability study based on site-directed mutagenesis showed low catalytic efficiency in the Gln27/Tyr70 variation, toward other haplotypes. These results confirm that patients carrying the mutant variant Tyr70 (208AA) may have a greater toxic exposure to cytarabine based therapy [206]. However, homozygous CDA Tyr70 variants very low frequent in Caucasian population [150].

Lately, beyond these recognized variants in CDA gene, other intronic polymorphisms were identified as involved in low enzyme efficiency in Asian populations. Among these the SNP 235-209T>C, detected in intron 3 at a frequency of 0.06, has value in pharmacogenomics [207].

CMPD deaminates cytarabine-monophosphate to arabinosyl uracil-monophosphate. Genomic screening of coding regions and the proximal promoter of CMPD in Caucasian and African ethnic groups identified a non-synonymous SNP 172A>G (Asn58Asp) with a significant loss of activity in vitro assays [205].

Functional genomics studies in NT5C2 have been identified over 41 SNPs by GWA studies on ethnically well-defined DNA. Poor mRNA expression was detected in the presence of C>T rs11598702 in NT5C2; in addition, this SNP occurred in linkage disequilibrium with rs1163238 and 11191612, two further SNPs located in 5′ UTR [151]. The real clinical relevance of these genetic polymorphisms is to be established. Among NT5C3 polymorphisms, the association of synonymous Tyr92Tyr (nucleotide 276T) and non-synonymous His283Asp is correlated with decreased level of enzyme activity. These findings suggest that a genetic variation in NT5C3 gene may influence drug response [152].

### 6.2. Deaminases, 5′-Nucleotidases and Response and Toxicity to Cytarabine

There have been several studies focusing on the influential effect of generic polymorphisms in the CDA genes on cytarabine pharmacokinetics and pharmacodynamics. Patients with impaired CDA activity might develop strong toxicities after administration of cytarabine while CDA overexpression in tumor tissues might reduce the antitumor efficacy of the drug [134]. The most significant influence between subject variability and toxicity has been observed in “poor metabolizer” CDA*2A and *2B variants (Supplemental Table S1). In patients with AML, elevated levels of CDA have been directly correlated with relapse and lower levels of CDA with prolonged remission [203,208].

A study performed on native PBMCs from 100 healthy volunteers reported a cytarabine-induced toxicity of approximately 53% among individuals carrying two *2A alleles that are higher than carriers of no *2A alleles and nearly 74% higher than carriers of two wild-type *1A alleles [16]. The most important association has been observed for *CDA*-*31Del*, where the CDA mRNA expression has been 1.37-fold increased among homozygote carriers of the deletion compared with wild type carriers (no deletion-31) [16].

CDA enzyme deficiency may occur in poor metabolizers patients carrying homozygous CDA 79C allele as assessed by different studies [10]. An increase in post induction treatment-related mortality for the C allele of the 79A>C variant has been reported among 457 children with AML receiving cytarabine within Children’s Cancer Group 2941 and 2961 protocols [209]. The rate of post-induction treatment-related death was 2.5-fold higher in the 79CC group as compared with children with two wild-type alleles [209]. Differently, a lower incidence of grade III and IV liver toxicity was recorded within the multicenter trial AML96 of the German Study Initiative Leukemia among patients carrying the wild-type alleles of the 79AA [191]. In this study polymorphism, −451TT was found as an additional single *CDA* variant of clinical prognostic relevance among the 360 AML adult patients receiving cytarabine, was associated with risk for death >50% as compared with wild-type carriers and maintained independent prognostic value also in multivariate analysis [191].

In both studies, only the influence of single *CDA* variants has been studied and no haplotype analysis has been performed.

The role of CMPD in the pathogenesis of toxicity of cytarabine, has been poorly defined differently from the congener analog gemcitabine whose enzymatic deamination activity is impaired in the presence of the SNP 172A>G (Asn58Asp) with consequent toxicity due to reduced clearance of monophosphorylated metabolites [205].

Since monophosphorilated intermediate of cytarabine activation is reduced by cytosolic 5′-nucleotidases NT5C2 and NT5C3, the activity level of this enzyme may represent one of the factors affecting the clinical outcome of cytarabine therapy. Increased expression of NT5C2 has been correlated with resistance to cytarabine chemotherapy and to a lower survival rate in a hundred patients undergoing cytarabine chemotherapy [210].

An increase in the NT5C2 has emerged as a mechanism of resistance to cytarabine. Patients with AML and low expression level of NT5C2 have a better overall survival after treatment with cytarabine than patients with high expression [211]. NT5C2 is implicated in pharmacokinetic of cytarabine has been associated with poor clinical outcome [151].

Further evidences for the implication of NT5C2 in the cellular response to cytarabine came from the correlation between the formation of cytarabine triphosphate in blasts from patients with AML and their corresponding ratio of the expressions of DCK and NT5C2 [212]. In a recent genetic association study of disease free survival in 154 AML patients of European ancestry, five SNPs in the NT5C2 region were predictive [96]. The aforementioned data have prompted a search for NT5C2 inhibitors whose structural optimization is currently ongoing [213]. High NT5C3 expression was found to be associated with better outcome for AML patients receiving cytarabine [210].

## 7. Drug Damage Repair

### 7.1. Genetic Variants in DNA Repair Genes

Pharmacogenomic studies in cancer cells have consistently shown increased activity of nuclear protein able to remove alien nucleotides from DNA [214]. DNA Repair mechanism is controlled essentially by the Base Excision Repair (BER) and Nucleotide Excision repair (NER) genes family; furthermore genetic variants in any of these genes may modulate repair capacity and contribute to individual variation in chemotherapy response. Primary genes involved in DNA adduct restoration are the X-ray Cross-Complementing group (XRCC) and Excision Repair Cross-Complementing group (**ERCC,** also named XPD). In addition, other genes as ATM, RAD51, and BRCA1 are described to be involved in resistance to nucleosides analog [215].

#### 7.1.1. DNA Repair Genes and Response and Toxicity to Cytarabine

Germline variation in DNA repair gene encoding XRCC1 codon Arg399Gln variants has been associated to decrease in risk of toxicity in cancer [216]. In addition, two variants in ERCC2 codon Lys751Gln and Asp312Gln were associated with better treatment outcomes in patients with AML receiving cytarabine based-therapy [153]. In a study of 307 AML adult an increased risk of relapse was associated to heterozygosis phenotype for Lys751Gln [217].

#### 7.1.2. ‘Out of Pathway’ Genes

Even if the genes or enzymes pathways which control cytarabine mechanism are known, these may or may not explain variation in response. So, while this knowledge has guided the initial molecular assays, subsequently, with the high-density genotype data available for the HapMap samples the application of genome-wide genotyping and whole-genome sequencing have led to the discovery of genes out of pathway showing correlation with phenotypes of toxicity and response to cytarabine, and representing potential novel pharmacogenomics markers.

Associations between the expression of two genes (*GIT1* and *SLC25A37*) and cytarabine were validated in an additional panel of LCLs.

GIT1 acts as a scaffold for certain intracellular signaling cascade proteins, including the MAP kinase pathway. Increased *GIT1* expression has been shown to increase MAP kinase signaling [218,219], which may result in increased apoptosis in response to cytarabine [163].

*SLC25A37* is a member of the SLC25 solute carrier family. This carrier imports iron into mitochondria and is involved in heme biosynthesis [220]. Interestingly, intracellular iron concentration has been shown to be related to cytarabine cytotoxicity. In a study of leukemia cell lines, exposure to desferrioxamine and therefore depletion of intracellular iron resulted in increased sensitivity to cytarabine [221].

This is also the case of genetic variation in *FKBP5* gene, a candidate gene outside the metabolic nucleoside analogue pathway whose down regulation showed reduced sensibility to cytarabine in two hundred ethnically defined Human Variation Panel lymphoblastoid cell [5]. The SNPs rs3798346 and rs7755289 in FKBP5 gene were associated with statistically significant worse survival in 187 pediatric AML patients [222].

Decreased expression of RAD51, would result in weakened repair of DNA double strand breaks induced by cytarabine triphosphate, thus reducing cell survival [223]. This mechanism has prompted the use of HDACIs sine these agents suppress the transcript and protein levels of the transcription factor gene E2F1, a key factor for RAD51 expression [223].

Recently, a GWA based study, involving 523 LCLs, identified several statistically significant SNPs in Mutated Colorectal Cancer (MCC) gene associated with cytarabine sensitivity. In particular, one of them the rs1203633 with AA genotype was linked with poorer OS (*p* = 0.015) in AML patients of multicenter AML02 trial.

## 8. Consistency and Applicability of Pharmacogenomic Studies on Cytarabine

Candidate gene approaches and GWA studies have found out some subsets of patients more likely to benefit from cytarabine-inclusive treatment or those more likely to experience adverse events. In particular, the expression levels or genetic polymorphisms of genes within the cytarabine biotransformation pathway including *SLC29A1, DCK, CDA, RRM1, NT5C2* has appeared promising predictors of prognosis or toxicity to this drug.

However, translating laboratory findings into the clinic has been painfully slow and the real contribution of genetic variation of these candidate genes to exposure and response to cytarabine is overall unsettled despite differences in outcomes and toxicities emerged from clinical trials [224,225,226]. Ideally, in both candidate gene and GWA studies, patients will have been treated within a single-agent study and not a combination one, so to attribute properly phenotypic effects or absence of an effect. Moreover, when associations between genetic polymorphisms and drug concentrations, response, or toxicity were detected, a replication study was required. Accruing large patient cohorts receiving the same drug regimen for GWA studies is challenging, and even more difficult may be performing a replication GWA in patients. Such replication studies should have been unconducted in subjects with analogous ethnic backgrounds since the relative impact of polymorphisms may differ depending on racial background. As a consequence, a large part of findings was identified retrospectively and presented some levels of biases; others were derived from relatively small-sized samples of patients and, though acquired prospectively, deserved confirmation in larger cohorts. The difficulty in conducting large prospective pharmacogenomic or pharmacoeconomic trials (preferentially through a phase III design or as independent study) has led in the past several years a large number of investigators to employ cell-based models as a component of their pharmacogenomic research program. Some researchers have used the International HapMap or the Polymorphism Discovery Panel LCLs, whereas other groups have created their own cell lines from individuals with a specific disease for study. Actually, with respect to cytarabine, stronger evidences for promising results and translational application come out when genetic variants observed in LCLs are replicated with clinical patient data as is the case of genetic variants of *NT5C2* [151] and *FKBP5* [222]. Anyway, discoveries made in LCLs also deserve to be verified for replication in clinical datasets, and this successfully happened in very few cases.

Methodological constraints for replication and validation studies still remain after the accessibility of genome sequence and introduction of the new analytical platforms repertoire including single nucleotide polymorphism arrays, gene expression profiling, and next generation sequencing.

Beyond the limitations due to the necessity of replication and validation studies, GWA approaches as issues and shortcomings strictly related to computational methodology and statistical analysis of the massive amounts of data achieved. These exceptional volumes of results are susceptible to an unprecedented potential for false positives because of the huge number of comparisons and statistical tests that are made using data on SNPs or expression across the genome [227]. Lack of well-defined case and control groups, insufficient sample size, control for multiple testing and control for population stratification are other common problems [227]. Due to inadequate sample extents, effect sizes are often biased upward with large standard errors, particularly in the case of SNPs with low minor allele frequencies [227]. Since these high-throughput genetic investigations have brought about new computational challenges in the analysis and ranking of discoveries intended to categorization of genetic loci potentially predictive of phenotype, collective expertise among statistic geneticists, genotyping laboratories, and clinical investigators is required for appropriate study setup and quality control. To partially obviate the heterogeneity of the results of GWA studies of cytarabine-induced cytotoxicity, meta-analysis was recently adopted by researchers [228]. Anyway, population differences and heterogeneity of hematologic tumors and other potential covariates, such as concomitant medications can confound pharmacogenomics correlations in pharmacogenomic studies combined in the meta-analysis. Tests of heterogeneity and watchful interpretation of final output should be adopted in these cases [229,230]. An additional resource may be represented by the use of Bayesian models which may allow also the sequential incorporation of new data as soon as they are produced, also from ongoing studies before ending [231,232].

Anyway, due to the rapidly decreasing price, the new technique of complete genome sequencing will provide in the next future a realistic alternative to genotyping array-based GWA studies and side-step some of the limitations of non-sequencing GWA [233].

With respect to economic costs, overviews of cost-effectiveness studies on pharmacogenetics and genomics technologies are now available [234,235], as well as solicitations from diagnostic advisory committee, such as the National Institute for Health and Clinical Excellence (NICE) to Pharma and Academic communities to output design and data sources in economic models of healthcare [236]. Anyway, only few studies have addressed the cost-effectiveness of pharmacogenomics testing implicated in clinical practice [235]. Platforms, such as the Affymetrix DMET chip and the Illumina ADME, allow a targeted evaluation of genes known to be related to cytarabine and phenotype of interest. Alternatively, a targeted exome or genome capture experiment could be considered if there is a list of genes hypothesized to be relevant. This latter approach will most likely only be relevant until the cost of whole-genome sequencing drops. Once the cost of sequencing the entire genome is low enough, this will be the method of choice as it enables one to obtain the rare variants as well as the common variants and everything in between.

However, the real impact of pharmacogenomics in predicting the response to cytarabine-containing treatments and, consequently, refining a risk stratification that would translate into effective targeted therapies is still far from being defined. Several factors should be considered in determining clinical and prognostic significance of a novel genetic discovery: treatment schedule and concomitant medication, independent verification in multiple prospective clinical trials, independent prognostic value in multivariate analysis with consolidated prognostic factors, and the importance of the novel genetic aberration, either as potential targets or as modifiers of specific therapies.

Additional constraints come from the necessity of new study design. Refining treatment stratification and defining subgroups for targeted treatments are just the tip of the iceberg of more complex reality we have to face in designing future studies. In particular, when adding a new factor in the study protocol, one may consider the real distribution of the factor in the patient population, the significance of its role in terms of prognosis in combination with other consolidated prognostic factors and the probability that changing risk group (and thus the treatment intensity) for some patients may actually ameliorate their outcome [237]. For example in schedule including a BCL2 inhibitor Venetoclax, protocol-recommended dose modifications (50%) for patients Poor metabolizers for CYP3A or patients receiving strong CYP3A inhibitors [38].

This evaluation should also be performed to avoid unnecessary complexity in the stratification system, which is relevant for trial design, feasibility, and generalizability of the results.

Adding a new stratification factor would be worthwhile only if this increased the separation between risk groups while decreasing the with ingroup heterogeneity in terms of outcome.

### 8.1. New Cytarabine Formulations

Beyond the use of genetic make-up of patients to predict response to treatment, the progress in pharmacogenomics has brought in-depth knowledge of the mechanism of action of cytarabine and prompted the development of new compounds that could play independently of membrane transporters or kinases and are less susceptible to degradation. Therefore, while the demonstration of improved diagnostic prediction of the aforementioned genetic variants and their translation into clinical practice keep on pending and represent a still slow process, new formulations and cytotoxic analogues of cytarabine have shown promise in in vitro and animal models so prompting therapeutic clinical trials [21].

Protection of cytarabine from fast degradation and elimination has been primarily investigated, with the aim to prolong exposure of cells to cytotoxic concentrations. Since cytarabine is an S-phase specific drug an extended exposure of the cell to the drug is critical to achieve maximum cytotoxic activity. After encapsulation into pharmaceutically acceptable carriers and derivatives, cytarabine cannot be deaminated and seems to exhibit better pharmacokinetic parameters. As a consequence, new nucleotides with modified base- and sugar-moiety, pronucleotides, chemo conjugated analogues and liposomal formulations are contender to replace cytarabine in clinical practice, and even broaden therapeutic indications.

Conjugation of cytarabine to phospholipids, steroids or fatty acids can modify the pharmacokinetics of the drug ensuring easier transmembrane uptake, independent from diffusion facilitating systems, and lower sensitivity to deactivation by CDA [238,239].

#### 8.1.1. Elacytarabine

Ela cytarabine and elainic-acid conjugated cytarabine, has shown to be able to bypass the effect of hENT inhibition on CCRF-CEM cell line and in patient samples [240]. In preclinical and clinical studies, elacytarabine has shown both safety and efficacy in AML, with remarkable activity among the cytarabine-refractory AML population [239]. Ela cytarabine has also shown safety in a phase III study enrolling 381 patients with AML, but the activity of elacytarabine was not predicted by hENT1 expression [241]. To date, no additional phase III trials was published after 2014.

#### 8.1.2. Sapacitabine

A recent phase II study has explored in AML patients safety and activity of sapacitabine, an analogue of cytarabine incorporating a cyano group capable of nucleophilic attack with consequent strand breaks once incorporated into DNA [242]. Investigations of sapacitabine have demonstrated a novel action mechanism of causing a single-strand nick after its incorporation into DNA [243]. Differently from cytarabine, which causes stalling of replication forks and arrest in S phase, the DNA breaks caused by sapacitabine activates the G2 checkpoint and cause an accumulation of cells in the G2 phase of the cell cycle [244,245]. However, single strand breaks induced by sapacitabine is contrasted by DNA repair complex genes that includes *XRCC3*, *BRCA2*, and *RAD51* in AML [215].

Phase II trial are now exploring sapacitabine activity in chronic lymphoid leukemia and cutaneous T-cell lymphomas (ClinicalTrials.gov identifiers: NCT01253460 and NCT00476554).

#### 8.1.3. Pronucleotides

Phosphorylated cytarabine analogues have been developed in which the phosphate moiety has been chemically masked so to increase hydrophobicity and facilitate entrance of the compound into the cell. The phosphodiester derivative of cytarabine UA911 has been able to circumvent DCK deficiency [246] while another pronucleotide, cytarabine phosphoramidate, has demonstrated better activity on hENT1- and DCK-deficient cells than cytarabine [247]. The addition of one or more S-acyl-2-thioethyl group to cytarabine monophosphate has been able, after the sequential actions of a non-specific esterase and a phosphodiesterase, to release into the cell the active metabolite and circumvent resistance mechanisms due to altered membrane transport or decreased DCK [248].

Activated metabolites of nucleoside analogs may also be conjugated to innovative polymeric nanogels. This formulation, beyond permitting oral administration due to its stability in the gastrointestinal tract, was capable of an efficient transport and intracellular delivery of active drug form and resulted in substantial tumor growth inhibition in drug-resistant tumor xenograft animal model [249].

#### 8.1.4. Liposomal Formulations

A liposomal sustained-release formulation for intrathecal administration of cytarabine is available for the treatment of lymphomatous meningitis; due to pharmacokinetic characteristics and half-life in CSF of 100–263 h protracted exposure to the drug may be achieved by cancer cells in liquor [25]. However, severe neurotoxicity has been reported in different retrospective studies. Transient chemical meningitis [250], permanent damages in about 10% of patients [251], exacerbations by concurrent administration of high-dose methotrexate [252,253] and the advice that any grade of adverse neurological events, that may affect the same group patients with all others completely free from any toxicity [254,255], suggest that a neurotoxicity to liposomal cytarabine may occur in an especially predisposed subset of patients.

The co-encapsulation of two or more synergic antineoplastic agents in the same liposomal device, that could serve as a springboard to reduce the effective drug dose and consequently soothe side effects, represents an innovative option in cancer therapy already adopted for improving the activity of pyrimidine analogues [256]. After positive results in an early-stage trial [257], the CPX-351, a liposomal multidrug carrier encapsulating cytarabine and daunorubicin, is under evaluation in children with relapsed acute myeloid leukemia (ClinicalTrials.gov identifier: NCT01943682) and adult patients with untreated myelodysplastic syndrome or acute myeloid leukemia at high risk of treatment-related mortality (ClinicalTrials.gov identifier: NCT01804101). This new combined formulation was FDA and EMA approved in 2018.

## 9. Conclusions and Future Outlook

The last decade has witnessed significant advances in genome-wide profiling technologies (e.g., microarray and the next-generation sequencing), thus opening up the possibilities for high-throughput profiling and measuring of various molecular targets including gene expression, genetic variations, and more recently, epigenetic variations. In the meantime, the launch of the Human Genome Project and other parallel large-scale projects such as the International HapMap Project [258,259] and the 1000 Genomes Project [260,261] has promoted an unprecedented improvement of our understanding of the human genetic variation from diverse global populations. Pharmacogenetic and pharmacogenomic studies on cytarabine have benefited from these technical advancements and scientific breakthroughs during the past decade.

The use in covert resistant diseases and the worrying profile of toxicities, even life-threatening, have made cytarabine as one of the antineoplastic drugs most studied by pharmacogenetics and pharmacogenomics. Exploring drug transport and cellular metabolism as key determinants of pharmacokinetics and pharmacodynamics, as well as heterogeneity in response and toxicity profiles [20] has led to find out genetic determinants of response and toxicity to cytarabine. Indeed, the potential of genetic testing in the setting of clinical trials and practice remains largely unexploited. Since straightness and certainty of results from standard and GWA studies deserve repetition and validation in prospective clinical trials, also powered by properly devised statistical designs and analyses, a huge extra-time seems to be necessary to ascertain whether this strategy will eventually result in safer and more effective personalized treatments. We can expect more and sooner from the introduction of engineered variants of cytarabine that can bypass some critical steps of the metabolism and optimize intracellular performance of the drug. Progress seems more substantial and within reach for these new formulations where the drug is conjugated with lipids, or assembled in activated form as pronucleotides, or encapsulated in liposomal single or multi drug carrier.

The crosswise examination of cytarabine-related issues proposed in this article, ranging from the spectrum of clinical activity and severe toxicities, through updated cellular pharmacology and drug formulations, up to the genetic variants associated with drug-induced phenotypes, has tried to arouse and maintain the interest of both basic researchers and the clinical hematologists. The opportunity of soothing or overcoming the serious adverse events related to cytarabine-based therapies and the hope of predicting response and circumventing drug-resistance phenomena can hopefully rely on the future implementation of the methods for genotyping the variants influencing cytarabine biotransformation, but above all on transversal cognizance and mutual confrontation about current boundaries of clinical practice and the multiplicity of the biological and translational acquisitions. Probably, more restrictive criteria for patient selection as well as further extension of the investigation to diseases other than AML, especially lymphoma, could eventually open up new opportunities and indications. A halfway acquaintance about the contests in treating patients and the exciting outlooks of genetic analysis would hopefully enhance investigation in leukemia and lymphoma patients of pharmacogenomics assumptions in clinical trials on cytarabine-centered therapies. If we take into account that mostly it deals with orphan diseases with not much room for large and well-powered studies, a greater awareness and a mutual encounter between geneticists and hematologists, prior to the involvement of statisticians and industry, represent the privileged way to connect the future of pharmacogenomics and outcome in leukemia and lymphoma patients even.

## Figures and Tables

**Table 1:** Overview of cytarabine dose intensities and schedules in regimens for acute myeloid leukemias in adults. Details concerning dosing in combination regimens should also be consulted.

Disease	Phase	Regimens	Cytarabine Dosing and Additional Drugs	References
Acute myeloid leukemia	induction	*7 + 3*	100 mg/m^2^/day continuous infusion for 7 days (in combination with daunorubicin or idarubicin or mitoxantrone) or (Adults < 60 years) 200 mg/m^2^/day continuous infusion for 7 days (in combination with daunorubicin)	[39,40,41,42]
		*Low-Dose SubQ*	Adults ≥ 65 years: SubQ: 20 mg/m^2^/day for 14 days out of every 28-day cycle for at least 4 cycles or 10 mg/m^2^ every 12 h for 21 days, or 10 mg/m^2^ every 12 h for 21 days; may repeat after 15 days	[28,29]
	consolidation	*5 + 2*	100 mg/m^2^/day continuous infusion for 5 days (in combination with daunorubicin or idarubicin or mitoxantrone)	[39,40]
		*5 + 2 + 5*	100 mg/m^2^/day continuous infusion for 5 days (in combination with daunorubicin and etoposide)	[43]
		*BCL2 inhibitor/LDAC 4 + 4*	Venetoclax once daily, began at 100 mg on day 1 and increased stepwise over 4 days to reach the target dose of 600 mg (100, 200, 400, and 600 mg); dosing was continued at 600 mg per day from day 4 through day 28 in combination with 20 mg/m^2^ of Ara-C	[38]
		*High-Dose single-agent*	Adults ≤ 60 years: 3000 mg/m^2^ over 3 h every 12 h on days 1, 3, and 5 (total of 6 doses); repeat every 28–35 days for 4 courses	[32]
		*Intermediate-dose*	cycle I: cytarabine 200 mg/m^2^ per continuous infusion on days 1–7; included idarubicin at 12 mg/m^2^ (3-h infusion on days 5, 6 and 7); cycle II: cytarabine 1000 mg/m^2^ intravenously for 3 h twice daily on days 1–6; included amsacrine 120 mg/m^2^ per 1-h infusion on days 3, 5 and 7	[37]
	salvage	*ADE*	100 mg/m^2^ I.V push every 12 h for 10 days (in combination with daunorubicin and etoposide)	[44]
		*CLAG*	2000 mg/m^2^/day over 4 h for 5 days (in combination with cladribine and G-CSF)	[45]
		*CLAG-M*	2000 mg/m^2^/day over 4 h for 5 days (in combination with cladribine, G-CSF, and mitoxantrone)	[46]
		*FLAG*	2000 mg/m^2^/day over 4 h for 5 days (in combination with fludarabine and G-CSF)	[47]
		*High-Dose*	3000 mg/m^2^ over 1 h every 12 h for 12 doses (± an anthracycline)	[31]
		*MEC*	1000 mg/m^2^/day over 6 h for 6 days (in combination with mitoxantrone and etoposide)or Adults < 60 years: 500 mg/m^2^/day continuous infusion days 1, 2, and 3 and days 8, 9, and 10 (in combination with mitoxantrone and etoposide)	[48,49]
Acute promyelocytic leukemia	induction	*APL2000* *C9710*	200 mg/m^2^/day continuous infusion for 7 days beginning on day 3 of treatment (in combination with tretinoin and daunorubicin)	[50,51]

Abbreviation: LDAC: low-dose Ara-C.

**Table 2:** Overview of cytarabine regimens including cytarabine for lymphoid neoplasms in adults. Details concerning doses in combination regimens should also be consulted.

Disease	Phase	Regimens	Cytarabine Dosing and Additional Drugs	References
Acute lymphocytic leukemia	induction	Hyper-CVAD	Dose-intensive regimen: I.V.: 3000 mg/m^2^ over 2 h every 12 h days 2 and 3 (4 doses/cycle) of even numbered cycles (in combination with methotrexate; alternates with Hyper-CVAD)	[54]
		*Larson regime SubQ*	Early intensification phase: 75 mg/m^2^/dose days 1 to 4 and 8 to 11 (4-week cycle; repeat once) Late intensification phase: 75 mg/m^2^/dose days 29 to 32 and 36 to 39	[67]
	induction, relapse or progression	*High-Dose*	3000 mg/m^2^ over 3 h daily for 5 days (in combination with idarubicin [day 3])	[68]
Chronic lymphocytic leukemia	refractory or Richter’s syndrome	*OFAR*	I.V.: 1000 mg/m^2^/dose over 2 h days 2 and 3 every 4 weeks for up to 6 cycles (in combination with oxaliplatin, fludarabine, and rituximab)	[69]
Burkitt and Burkitt-like lymphoma	induction	*CALGB 9251*	Cycles 2, 4, and 6: 150 mg/m^2^/day continuous infusion days 4 and 5	[52,70]
		*CODOX-M/IVAC*	Adults ≤ 65 years: Cycles 2 and 4 (IVAC): 2000 mg/m^2^ over 3 h every 12 h days 1 and 2 (total of 4 doses/cycle) (1000 mg/m^2^ if age > 65) (IVAC is combination with ifosfamide, mesna, and etoposide; IVAC alternates with CODOX-M)	[53]
Mantle cell lymphoma	induction	*R-BAC*	cytarabine 800 mg/m^2^ IV on days 2 to 4) every 28 days for four to six cycles (in combination with rituximab and bendamustine)	[57]
CNS lymphoma, primary	induction		I.V.: 2000 mg/m^2^ over 1 h every 12 h days 2 and 3 (total of 4 doses) every 3 weeks (in combination with methotrexate and followed by whole brain irradiation) for a total of 4 courses	[66]
Hodgkin and non-Hodgkin lymphoma	relapse or progression	*DHAP*	2000 mg/m^2^ over 3 h every 12 h day 2 (total of 2 doses/cycle) for 2 cycles (in combination with dexamethasone and cisplatin)	[62,71]
		*ESHAP*	2000 mg/m^2^ day 5 (in combination with etoposide, methylprednisolone, and cisplatin) every 3 to 4 weeks for 3 or 6 cycles	[60,63]
		*DHAOX*	2000 mg/m^2^ every 12 h day 2 (in combination with dexamethasone, and oxaliplatin) every 3 weeks	[61]
		*BEAM*	transplant preparative regimen: 200 mg/m^2^ twice daily for 4 days beginning 5 days prior to transplant (in combination with carmustine, etoposide, and melphalan)	[64]

**Table 3:** Essential features of cytarabine pharmacology.

Factor	Result
Mechanism of action	The active form, cytarabine triphosphate, induces miscoding after incorporation into DNA, terminates DNA chain elongation and inhibits DNA polymerase
Cellular influx and transporters	Free diffusion into the cell if plasma concentration >10 μM which is achieved with high-dose cytarabine Nucleoside transporters required if plasma concentration <1 μM which is achieved with 100–200 mg/m^2^ daily (hENT1 responsible for up to 80% influx; hENT2, hCNT3, ABCC10 and ABCC11 transporters also involved)
Metabolism enzymes	Stepwise phosphorylation of cytarabine in tumor cells at the 5′position of arabinoside up to triphosphate form by Deoxycytidine kinase (→cytarabine monophosphate) Deoxycytidine momophosphate kinase (→cytarabine diphosphate) Nucleoside diphosphate kinase (→cytarabine triphosphate) Irreversible deamination of cytarabine and its monophosphate intermediateinto inactive derivatives by Cytidinedeaminase (cytarabine→uracilarabinoside) Deoxycytidine momophosphate deaminase (cytarabine monophosphate →arabinosyluracil monophosphate) Catalytic dephosphorilation of cytarabine monophosphate by Cytosolic5′-nucleotidase II(cytarabinemonophosphate→cytarabine)
Pharmacokinetics	Plasma t ½α 7–20 min, t ½β 1–3 h; CSFt ½ 2–6 h
Elimination	Dose rate ranging from 86% to 96% is deaminated to inactive uracil arabinoside Deamination in liver, plasma, and peripheral tissue Intrathecal administration results in little conversion to uracil arabinoside due to the low level of deaminase in the cerebral spinal fluid Escretion: urine (~80%; 90% as metabolite uracil arabinoside) within 24 h
Drug interactions	Methotrexate and fludarabine increase cytarabine triphosphate formation Cytarabine blocks DNA repair and enhances activity of alkylating agents
Myelosuppression	Neutropenia (onset: 1–7 days; nadir [biphasic]: 7–9 days and at 15–24 days; recovery [biphasic]: 9–12 days and at 24–34 days); thrombocytopenia (onset: 5 days; nadir: 12–15 days; recovery 15–25 days); megaloblastosis
Severe adverse events of high dose regimen	Neurologic: cerebellar toxicity, coma, personality changes, cognitive dysfunctions, neurotoxicity (up to 55% in patients with renal impairment), motor and sensor peripheral neuropathy Gastrointestinal: esophageal and intestinalulceration, bowel necrosis, hepatic sinusoidal obstruction syndrome, pancreatitis, pneumatosis cystoides intestinalis Cutaneous: skin eruptions and ulceration, exanthematous pustulosis Ocular: vision loss, keratitis, hemorrhagic conjunctivitis Cardiopulmonary: non-cardiogenic pulmonary edema, syndrome of sudden respiratory distress, interstitial pneumonitis, cardiomiopathy (in combination with cyclophosphamide) Sepsis

**Table 4:** Mechanisms of resistance within the cytarabine pathway.

1. Deficiency of cytarabine cellular uptake and retention from
– decreased numbers or low activity of nucleotide transporters, particularly the transporter hENT1
– high activity of ABCC10 (MRP7) and ABCC11(MRP8) efflux pump
– reduced phosphorylation due to low DCK, CMPK and NDK enzymes levels or activity;
2. Overexpression of enzymes inactivating cytarabine, primarily CDA, CMPD and NT5C2
3. Increased cellular dCTP pools following overexpression of RNR or CTPS and followed in turn by
– antagonism for DNA incorporation of cytarabine-triphosphate
– inhibition, through a feedback mechanism, of DCK-catalyzed phosphorylation of cytarabine
4. Altered DNA polymerase and increased DNA repairgenes i.e., XRCC and ERCC groups.

**Table 5:** Overview of gene variants that may affect response to cytarabine.

	GENE (Protein)	DNA Variants			Minor Allele Frequency *			Phenotype		Activities Related SNP	Ref.
		rs #	Nucleotide	Location (Codon)	Afr	Eur	Asn	*Clinical*	*Cell Model*		
**Transport**	***SLC29A1*** **(hENT1)**	rs747199	−706G>C	5′UTR	(C) 0	(C) 0.21	(C) 0.23		increase in hENT1 mRNA expression in PBMCs and cytarabine uptake		[145]
	***SLC28A3*** **(hCNT3)**	rs11140500	T>C	5′UTR	(T) 0	(T) 0.01	(T) 0.29				[96]
**Activation**	***DCK*** **(DCK)**	NA/ rs2306744	−360C>G/−201C>T	5′UTR	NA	NA	NA	mRNA expression −360GG/−201TT haplotype shown good clinical response in AML	altered enzyme kinetics, increased cytarabine triphosphate intracellular concentrations		[146]
		rs66878317	70A>G	Exon 1 (Ile24Val)	(G) 0	(G) 0	(G) 0.03		Altered substrate kinetics		[147]
		rs67437265	364C>T	Exon 3 (Pro122Ser)	(T) 0.06	(T) 0.01	(T) 0.04		variant Pro122 shown lower enzyme activity		[147]
	***NDK-NME1*** **(NDK)**	rs2302254	835C>T	5′UTR	(T) 0.37	(T) 0.18	(T) 0.25	−835 T/T increased risk of neurotoxicity.			[148]
	***RRM1*** **(RRM1)**	rs1561876	−2993A>G	3′UTR	(G) 0.72	(G) 0.12	(G) 0.21	cytarabine triphosphate levels Increased, response and survival improved			[149]
**Deactivation**	***CDA*** **(CDA)**	rs532545	−451C>T	5′UTR	(T) 0.07	(T) 0.32	(T) 0.13	Decreased expression of CDA	PBMC from healthy donors, report that among individuals carrying two *2A alleles (seeTable 1), cytarabine-induced toxicity was approximately 53% higher whencompared with carriers of no *2A alleles and nearly 74% higher compared with carries of two wild-type *1A alleles.	Low mRNA level. High level of cytarabine triphosphate resulting in bone marrow depression	[16]
		rs602950	−92A>G	5′UTR	(G) 0.07	(G) 0.32	(G) 0.13				
		rs3215400	−31Del C	5′UTR	(C) 0.33	(C) 0.43	(C) 0.44	del/del shown 1.37fold increased expression than ins C/insC Parmar			
		rs2072671	79A>C	Exon 1 (Lys27Gln)	(C) 0.08	(C) 0.33	(C) 0.13	Low enzyme activity in Lys27Lys than Gln27Gln	Cytidine is the 2.4 fold lower K_m_ for Lys27Lys than Gln27Gln in European		[150]
		rs1048977	435T>C	Exon 4 (Thr145Thr)	(T) 0.37	(T) 0.33	(T) 0.25	Haplotype CDA *2A(435A) display higher CDA activity compared with others			
	***NT5C2*** **(NT5C2)**	rs11598702	175+1178A>G	5′UTR	(C) 0.19	(C) 0.35	(C) 0.22	Low mRNA expression and cytarabine sensitivity in cells derived from AML patients	Low mRNA DFS 17.5 months vs 11 months in AML	Low enzyme activity	[151]
	***NT5C3*** **(NT5C3)**	rs3750117	276T>C	Tyr92Tyr	(T) 0.17	(T) 0.29	(T) 0.5		In PBMCs haplotypes T276/H283 and T276/C306 decreased enzyme activity		[152]
**DNA repair**	***XRCC1*** **(XR_)**	rs25487	28152G>A	Exon (Arg399Gln)	(T) 0.12	(T) 0.35	(T) 0.25	Mutations are correlated to prediction of better treatment outcomes in patients with AML		Low activity in Base excision DNA repair	[153]
	***ERCC2*** **(XDP)**	rs13181	35931A>C	Exon (Lys751Gln)	(G) 0.21	(G) 0.38	(G) 0.09			Low activity in Nucleotide excision DNA repair	[153]

Abbreviation: NA, not assigned. * data from Ensamble (www.ensembl.org/Homo_sapiens/Variation/Population?db=core;r=9:22125003-22126003;v=rs1333049;vdb=variation;vf=1 (accessed on 13 January 2021)). # genetic locus.

## Data Availability

Not applicable.

## Supplementary Materials

The following are available online at https://www.mdpi.com/2072-6694/13/5/966/s1, Supplemental Table S1: CDA haplotype nomenclature.
