# Supplementary material for: Response and Toxicity to Cytarabine Therapy in Leukemia and Lymphoma: From Dose Puzzle to Pharmacogenomic Biomarkers
Source: Cancers (Basel). 2021 Feb 25;13(5):966. doi: 10.3390/cancers13050966 (PMC7956511; doi:10.3390/cancers13050966)
Supplement: Supplementary file 1 [file cancers-13-00966-s001.pdf]

Table S1. CDA haplotype nomenclature**.** Letters in bold denote variant allele.

Nucleotide Position	CDA Haplotypes **								
	* 1A	* 1B	* 1C	* 1D	* 1L	* 1Q	* 2A	* 2B	* 2D
−451C>T	C	C	C	C	**T**	C	**T**	**T**	C
−92A>G	A	A	A	A	**G**	**G**	**G**	**G**	A
−31Ins/Del	Ins	**Del**	**Del**	I	**Del**	**D**	**D**	**D**	I
79AC>T	A	A	A	A	A	A	**C**	**C**	**C**
435C>T	C	C	T	T	C	C	**T**	C	**T**
Frequency	0.47	0.03	0.14	0.02	0.01	0.01	0.13	0.21	0.01

* Nucleotide Position, ** from Gilbert JA et AL. Clinical cancer Research 2006,12,17942–803. In bold are reported minor allele polymorphisms.
